# Ordered Peierls distortion prevented at growth onset of GeTe ultra-thin films

**DOI:** 10.1038/srep32895

**Published:** 2016-09-09

**Authors:** Ruining Wang, Davide Campi, Marco Bernasconi, Jamo Momand, Bart J. Kooi, Marcel A. Verheijen, Matthias Wuttig, Raffaella Calarco

**Affiliations:** 1 Paul-Drude-Institut für Festkörperelektronik, Hausvogteiplatz 5-7, 10117 Berlin, Germany; 2 Department of Materials Science, University of Milano-Bicocca via R. Cozzi 55, 20125 Milano, Italy; 3 Zernike Institute for Advanced Materials, University of Groningen, Nijenborgh 4, 9747 AG Groningen, The Netherlands; 4 Eindhoven University of Technology, Department of Applied Physics, NL-5600 MB Eindhoven, The Netherlands; 5 I. Physikalisches Institut, RWTH Aachen University, 52056 Aachen, Germany; 6 JARA-FIT and JARA-HPC, RWTH Aachen University, 52056 Aachen, Germany

## Abstract

Using reflection high-energy electron diffraction (RHEED), the growth onset of molecular beam epitaxy (MBE) deposited germanium telluride (GeTe) film on Si(111)-(√3 × √3)R30°-Sb surfaces is investigated, and a larger than expected in-plane lattice spacing is observed during the deposition of the first two molecular layers. High-resolution transmission electron microscopy (HRTEM) confirms that the growth proceeds via closed layers, and that those are stable after growth. The comparison of the experimental Raman spectra with theoretical calculated ones allows assessing the shift of the phonon modes for a quasi-free-standing ultra-thin GeTe layer with larger in-plane lattice spacing. The manifestation of the latter phenomenon is ascribed to the influence of the interface and the confinement of GeTe within the limited volume of material available at growth onset, either preventing the occurrence of Peierls dimerization or their ordered arrangement to occur normally.

Since the late 60’s, GeTe has attracted a lot of attention both from a fundamental and technological perspective; it has been kept under constant scrutiny as a thermoelectric^1^,^2^, ferroelectric^3^,^4^,^5^, and phase-change material^6^,^7^,^8^. Recently, its broad repertoire of known properties has been expanded with the prediction^9^ and demonstration^10^ of a giant bulk Rashba effect that paves the way towards novel spintronic devices. Such a rich array of functionalities in GeTe could come as quite a surprise considering the simple two-atom rhombohedral unit cell that is commonly used to describe it. In reality, GeTe is more complex than what it seems; the minimalistic ball-and-stick models are inadequate to fully describe the subtleties behind its structure. Namely, the interplay between a rhombohedral distortion caused by the electronic Peierls instability of a cubic material^11^, 6-fold coordinated resonant bonding in violation of the octet rule^12^,^13^, and no less than 10% of intrinsic Ge vacancies in the Ge sublattice^14^. Throughout literature, a consensus has now been reached that these three phenomena listed above are the main actors shaping the structure and defining the many properties of GeTe. More specifically, the rhombohedral distortion and spontaneous electric polarization of α–GeTe have been ascribed to an ordered Peierls dimerization^15^, the crystalline/amorphous switching mechanism has been ascribed to the formation and disordering of the 6-fold resonant bonded network^16^,^17^. As for the abundant intrinsic Ge vacancies, they have been shown to be responsible for the p-type metallic conduction, whereas the non-defective GeTe band structure is theoretically predicted to have its Fermi level in the bandgap^18^,^19^,^20^. In the present work, the focus is set on the lattice distortion induced by Peierls distortion in ultra-thin GeTe films grown by MBE and investigated by RHEED, Raman spectroscopy, and HRTEM.

As demonstrated previously^21^, when GeTe is grown on the fully passivated Si(111)-(√3 × √3)R30°-Sb surface, a crystalline phase is immediately formed at the very beginning of the growth, as opposed to growth on non-fully passivated surfaces such as Si(111)-(7 × 7) where the formation of an amorphous phase is initially observed^22^. In Fig. 1, RHEED data acquired during the early stages of GeTe growth on the Si(111)-(√3 × √3)R30°-Sb surface is presented. The right hand side of Fig. 1(b) shows the evolution of the RHEED intensity during deposition, acquired along a line across the RHEED streaks, as illustrated by the vertical dashed line across the left hand side RHEED image. Note that the α-GeTe unit cell is described with cubic notation, despite the trigonal distortion of the cubic rocksalt structure in the GeTe(111) direction. The data presented here is acquired along the Si< 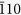 > azimuth, measuring the d-spacing between 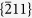 planes, as depicted in (a).

As soon as the deposition starts, at t = 0 s, new streaks appear immediately, while the streaks corresponding to the silicon substrate disappear within the first 10 seconds. Intensity oscillations can be measured by integrating the intensity of the specular beam as shown in Fig. 1(c). Following an abrupt decrease of intensity due to a transient change of surface conditions, a first minimum is found at t = 50 s, a maximum at t = 110 s, and a second maximum at t = 190 s. In a simplified model, these oscillations can be explained by a change in diffuse scattering caused by the increase and decrease of island step edges perimeter as the coverage increases^24^. The period of RHEED oscillations can therefore be used as a measurement of the growth rate, which is estimated at about one GeTe bilayer (BL) per 100 seconds (1 GeTe BL = 0.35 nm). A rapid damping of the RHEED oscillations is observed after two oscillations, showing that the layer by layer growth process is imperfect, additional layers nucleate and coalesce at the same time, before the full completion of the layers below.

The lattice spacing in the freshly deposited film can be calculated from the measured RHEED streak spacing, using the known lattice spacing of the silicon substrate as a reference. The evolution of the lattice spacing during growth is reported in Fig. 1(d). Immediately at growth onset, a {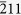} lattice plane spacing of 2.46 Å is measured, which is larger than the expected value of 2.41 Å for bulk rhombohedrally distorted α-GeTe (orange dashed line)^25^. This larger lattice spacing found at growth onset cannot be explained by an ordinary mismatch relaxation process, because relaxation would occur from the much smaller lattice spacing of the silicon substrate (2.22 Å). A comparison with literature shows that this initial value matches better with the cubic phase of β-GeTe at 2.446 Å as highlighted by a grey horizontal dashed line in Fig. 1(d) 25, or with metastable rocksalt Ge_2_Sb_2_Te_5_ (GST225) at 2.46 Å^26^, In any case, growth seems to be initiated with the formation of a phase different than the intended α-GeTe. Synchronized with the first minimum in RHEED intensity at t = 50 s, the lattice spacing then starts to drop, until the expected value for α-GeTe is reached around t = 200 s, with the completion of the second GeTe BL. The possibility for this phenomenon to be a kinetic effect is also ruled out by performing a similar growth experiment with a reduced growth rate. The exact same changes were observed, but at a slower rate. Details for this slower growth are presented in the Supplementary Information along with Figure S1. Furthermore, the same experiment is repeated in order to monitor the RHEED pattern of the other in-plane orthogonal 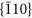 lattice planes (not shown here). The same behaviour is observed, with an initial lattice spacing 2.2% larger, that shrinks back to the expected value for α-GeTe within the first 200 s of growth.

Finally, another point that should be addressed at this point is the apparent discrepancy between the growth rate calculated from RHEED intensity oscillation and the rapid fading of the substrate RHEED streaks upon deposition. This is a particularly important matter in the present case because the substrate Si(111)-(√3 × √3)R30°-Sb reconstruction is a fully passivated surface, that is not expected to be lost upon deposition. It would therefore be reasonable to suppose the starting surface to be visible by RHEED until the completion of the very first GeTe layer. However, the optimal growth window utilized in these experiments involves high Te fluxes and high substrate temperature, as described in the methods section. This means that the film is grown in a regime where the whole surface is subjected to an important exchange of adsorbing and desorbing Te and GeTe. Meanwhile, the coverage of the coalescing crystalline GeTe islands is only increasing slowly. The rapid accumulation of this gaseous layer could be the reason why substrate streaks seem to fade so prematurely.

To further investigate the growth onset of GeTe on the Si(111)-(√3 × √3)R30°-Sb surface, very thin films of a few atomic layers were grown with nominal thicknesses of 0.5, 1, 2, 4, 6, 8, and 16 BLs. Until each growth is interrupted, the larger in-plane lattice spacing on the Sb terminated surface was consistently observed, testifying the reproducibility of this phenomenon. Moreover, as the growth is interrupted, the RHEED image is frozen and does not change further after the deposition is stopped, demonstrating the stability of these thin layers, and further showing that the phenomenon observed is not a kinetic effect.

TEM (Fig. 2) is performed on the GeTe film with 1 BL nominal thickness to corroborate the RHEED data. Already from a low resolution TEM (Fig. 2a), the film can be recognized by the strong dark line generated by the contrast in density between the film and the substrate. This micrograph demonstrates that the film is fully wetting at this stage, as expected from the complete disappearance of the silicon RHEED streaks upon growth. This demonstrates the capability of creating ultra-thin crystalline and wetting GeTe layers using MBE. Precautions should be taken in the interpretation of this first micrograph, as the thickness of the layer does not equate the thickness of the dark features it generated. A higher resolution micrograph along the Si< 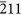 > direction (Fig. 2b) reveals that there is crystalline order within the film, as it will be further demonstrated by Raman measurements. There is also evidence for the imperfect layer by layer growth, with the nucleation and coalescence of several molecular layers at the same time, causing the rapid dampening of the RHEED intensity oscillations. It is important to highlight that each sample is capped with 10 nm of Si_3_N_4_ prior to extraction from the MBE system, to protect the ultrathin films from oxidation. While it is difficult to assess the exact effect of the capping on such an ultrathin film, crystalline order could still be observed, therefore we may assume it did not drastically alter the material.

Raman spectroscopy is then performed on each of these GeTe films, the measurements are acquired in z(y,xy)-z geometry with a 633 nm laser and shown in Fig. 3. The features at 225 cm^−1^ and 300 cm^−1^ respectively, which are visible in the silicon reference spectrum, correspond to the second order scattering by transverse acoustic phonon modes 2TA(L) and 2TA(X) of silicon (highlighted by grey arrows)^27^. With increasing thickness of the GeTe film, these modes from the silicon substrate become less intense. Except from those modes, only a weak and broad feature can be measured at 150 cm^−1^ in the two thinnest samples. This only Raman feature is not related to the GeTe film, but originates from the ultra-thin Sb layer passivating the surface^28^. In stark contrast, two much clearer features can be resolved for the thicker samples. While they are clearly evidenced in the 4 BL sample, fitting by three Gaussian functions reveal that these modes are also present in the 2 BL sample as well, as shown in the magnified plot of Figure S2 in Supplementary Information. Both peaks identified as the (E) and (A_1_) modes of GeTe^29^ at 80 cm^−1^ and 120 cm^−1^ respectively are subjected to a thickness related mode strengthening similar to what has been observed for GeTe nanocrystals of increasing size^30^. The extent of the shift is plotted in Fig. 3(b). This clear difference between the spectra for samples below and above 4 BL could be explained by a lack of rhombohedral distortion and a suppression of Peierls distortions in the thinner samples. Indeed, the undistorted GeTe crystal tends toward a cubic rocksalt structure, in which case no first order Raman modes are expected^29^,^31^.

Because of the involvement of Sb in the surface preparation, intermixing with GeTe into GST has to be assessed as a potential cause for the larger lattice spacing observed at growth onset. It should be also noted that in the Raman spectrum of the 4BL sample shown in Fig. 3(a), the peaks at 110 cm^−1^ and 155 cm^−1^ coincide with the Raman spectrum of metastable GST^32^. To this effect, preliminary STEM micrographs in HAADF mode are shown in Figure S3 in the supplementary section. Ongoing efforts are invested in imaging these ultra-thin GeTe layers that are easily altered during preparation, or just over time due to oxidation^33^,^34^. Nevertheless, current results already show a strong bright line laying at the interface, demonstrating that the initial Sb-passivation is vastly left intact. Indeed, these Sb atoms are covalently bound to the silicon surface^35^, breaking these covalent bonds is expected to be energetically very expensive. For instance, the stability of this surface passivation can be appraised by considering that the substrate needs to be heated to 650–880 °C in order to fully desorb the monolayer of Sb absorbed on the Si substrate^36^. Therefore, also for the formation of an energetically favourable GST compound, a high barrier needs to be overcome. For instance, it has been previously shown that this Sb passivation is able to retain its stability, even after annealing at 400 °C in direct contact with an environment rich in Ge, Sb, and Te^37^.

Furthermore, if Sb atoms are removed and silicon bonds are somehow left unpassivated, growth of GeTe could be expected to yield in-plane twist domains, as it has been shown for GeTe grown on a partially unpassivated surface such as Si(111)-(7 × 7)^22^. This reconstruction leaves 19 dangling bonds per unit cell of 49 surface atoms, which means that 39% of the upward bonds are left unpassivated^38^. Because no pronounced in-plane twist domains are observed when growing GeTe on the Sb passivated surface^21^, it suggests that the surface remains widely passivated.

As the formation of a GST phase is ruled out, the shift of the Raman peaks should be carefully explained. To this end, we computed phonons at the Γ-point of GeTe multilayers by means of Density Functional Perturbation Theory (DFPT)^39^. Due to the mismatch in the lattice parameter between GeTe and the Si(111) surface, the commensurate surface cell of GeTe multilayers grown on Si(111)-(√3 × √3)R30°-Sb^21^ is too large to be addressed by DFPT methods. Therefore, in order to model the growth of GeTe multilayers on the Sb-passivated Si surface we actually considered a thick slab of GeTe with a number of layers free to move and few bottom layers frozen mimicking the surface substrate. The in-plane lattice parameter is fixed to the theoretical bulk value. For the thinner multilayers we also considered the in-plane lattice parameters fixed to the experimental value as measured during the MBE growth. The position of the Raman peaks calculated theoretically for the bulk at zero temperature with a concentration of holes comparable with experiments^40^ are at 84 cm^−1^ and 130 cm^−1^. Those values are plotted in Fig. 3(b) with empty symbols. The agreement with experiments is good taking into account that at room temperature we expect a red shift of the frequency computed at zero temperature^29^. The interface with the capping layer is also not taken into account in the simulation, and might contribute to the shift of the modes as well.

The DFPT Raman spectrum in backscattering configuration for non-polarized light is reported in Fig. 3(c) for the supported 4 BL (open symbols). The displacement patterns of the modes mostly contributing to the peak at 120 and 164 cm^−1^ are given in of Fig. 3(d). The spectrum compares well with the experimental spectrum once the redshift due to temperature, not included in our calculations, is considered. Then we considered configurations in which the free layers are shifted in the surface plane with respect to the bottom frozen layers (AB–AB–CA–BC–) in order to destroy the resonant bonding and reduce the coupling between the free layers and the frozen substrate. The effect on phonon frequency for the 4 BL is actually marginal (<2 cm^−1^).

On the other hand, the frequency of the A_1_ and E modes for the supported 1 BL and 2 BL are in the range 136–140 cm^−1^ and 184–195 cm^−1^ with variations depending on the choice of the in-plane lattice parameters (experimental or bulk-like) and the stacking of the frozen layers with respect to the free ones; modes in these latter frequency ranges have no experimental counterpart in the Raman spectrum. Thus the DFPT results corroborate the observation of the formation at growth onset of a phase different than α-GeTe which turns into the α phase.

Dispelling a common misconception, Gaspard *et al*. have predicted that crystalline periodicity is not necessary for Peierls distortions to occur^11^, the most convincing evidence supporting this claim is that Peierls distortions were observed in liquid GeTe close to the melting point^41^. Therefore the lack of out-of-plane long range periodicity in a very thin GeTe film should *a priori* not inhibit Peierls distortions, but the unusual boundary conditions with the presence of the interfaces with substrate and capping layer could still prevent Peierls distortions from being expressed normally. And even if Peierls distortions do occur, a high degree of ordering of the short and long bonds is needed in order to observe a net deformation of the overall crystal structure. If short and long bonds are disorderly distributed, the crystal will be seen as being cubic in average. From a theoretical standpoint, Gaspard *et al*. have also predicted that the ordering of the bonds was energetically favorable in the idealized case^11^. But experimental data shows that these bonds can be found in a disordered configuration: For instance, the initial belief that the rhombohedral to cubic transition from α–GeTe to β–GeTe was purely of a displacive nature^25^ has been more recently challenged^42^. The more recent extended X-ray absorption fine structure data could only be conciliated with the previous X-ray diffraction (XRD) data using an order-disorder transition model. A similar interpretation was also given by Biquard *et al*. in the case of nitrogen and carbon doping into GeTe, which would cause the loss of ordering of the Ge–Te bonds, such that the XRD spectrum would seem to correspond to a cubic structure by an averaging effect^43^. In the present case, because of the influence of the interface and surface, a certain degree of disorder in the bonding could be induced in the very thin films, making the in-plane lattice spacing larger in average. Then, as the growth continues, these bonds with the completion of the second GeTe BL have the chance to reorganize towards the more favorable ordered configuration.

To conclude, an in-plane lattice spacing larger than expected when depositing GeTe on Si(111)-(√3 × √3)-Sb was observed. The non-viability of Peierls distortions or the lack of their coherent ordering are designated as the fundamental physical mechanism responsible for the apparent larger in-plane lattice spacing in such ultra-thin films. If Peierls distortions cannot be fully manifested in an orderly fashion within the constrained volume at growth onset, the GeTe crystal may only acquire its ferroelectric properties as growth proceeds, once the bonds are Peierls-dimerized and ordered into layers. Considering the peculiar link between ferroelectricity and giant Rashba spin splitting in this material^9^,^10^, this could translate into a thickness dependence of the Rashba effect, analogous to the thickness dependence of topological insulator properties of ultra-thin Bi_2_Se_3_^44^. For scaling purposes, this suggests that special care should be taken in the selection of the bottom electrode, as the interface will play a decisive role for the ferroelectric properties of the ultra-thin film.

## Methods

All the substrates are cleaned using the methods described in Wang *et al*.^21^. The preparation of the Si(111)-(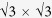) R30-Sb surface is also described in the cited publication. The growth itself is performed at a substrate temperature of 260 °C, using Ge and Te dual-filament effusion cells with base and tip temperature of T_base_(Ge) = 1120 °C and T_tip_(Ge) = 1140 °C for the Ge cell, T_base_(Te) = 335 °C and T_tip_(Te) = 470 °C for the Te cell. The cell fluxes are beforehand calibrated by performing X-ray reflectivity measurements on amorphous Ge and Te films grown at room temperature (RT). The cell temperatures used presently correspond to RT deposition rates of 0.17 nm/min for Ge and 0.4 nm/min for Te, resulting in a Ge/Te flux ratio of ~2/5. It has to be noted that RT deposition rates and growth rate differ greatly because GeTe desorption is highly relevant at the deposition temperature^45^. At the end of the growth, the sample is cooled down to room temperature, and prior to its removal from the MBE system, the sample is capped with 10–15 nm of Si_3_N_4_ by radio-frequency sputtering in the MBE load-lock chamber in order to prevent oxidation.

Cross-sectional TEM specimen were prepared along Si<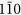 > or < 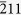 > crystallographic directions by mechanical polishing, dimple grinding and low-voltage Ar^+^ ion milling for final thinning using a Gatan PIPS II. TEM measurements were carried out on a JEOL 2010F and a JEOL 2010 and preliminary STEM measurements were carried out on a JEOL ARM200F. Calibration of images is typically performed on the basis of the Si(111) interplanar distance (0.3135 nm). Image analysis was carried out using GMS software.

Calculations were performed within the framework of density functional theory (DFT) with exchange and correlation energy functional proposed by Perdew-Becke-Ernzerhof^46^ and norm conserving pseudopotentials, as implemented in the *Quantum-Espresso* suite^47^. Only outermost s and p electrons were considered in the valence. Kohn-Sham orbitals were expanded in a plane waves basis up to a kinetic cutoff of 35 Ry. Brillouin Zone integration was performed over a 12 × 12 × 1 Monkhorst-Pack mesh^48^. Additional details are provided in the Supplementary Information section.

## Additional Information

**How to cite this article**: Wang, R. *et al*. Ordered Peierls distortion prevented at growth onset of GeTe ultra-thin films. *Sci. Rep.* **6**, 32895; doi: 10.1038/srep32895 (2016).

## Supplementary Material

Supplementary Information

## Figures and Tables

**Figure 1:**
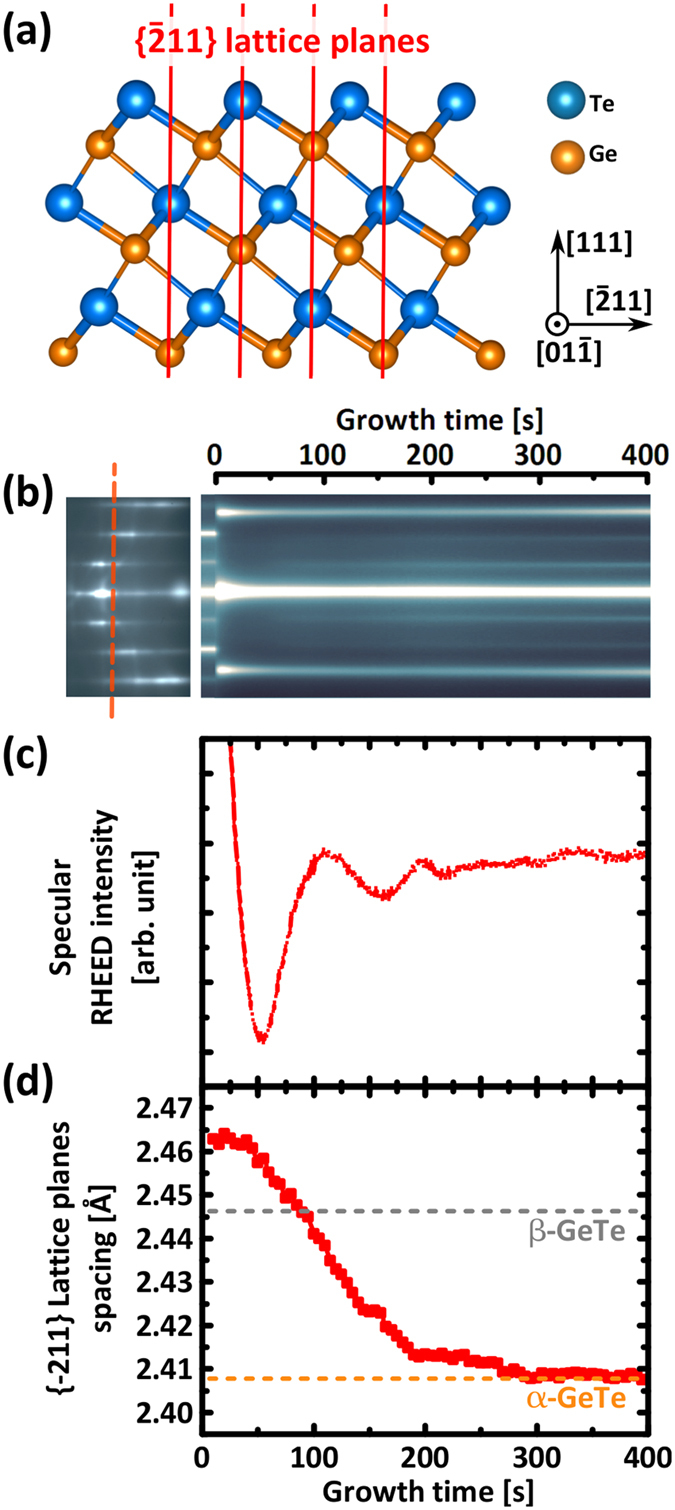
(**a**) Schematic model of α–GeTe crystal with periodic stacking of Ge and Te layers in the [111] direction. The (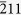) lattice planes of interest are highlighted in red. Scale models were created using VESTA software^23^. (**b**) RHEED intensity over time acquired along red dashed line across the < 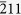 > azimuth RHEED pattern. (**c**) Integrated specular beam intensity oscillations close to growth onset demonstrating the formation of closed layers. (**d**) 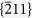 lattice planes spacing calculated from RHEED streak spacing showing a larger in-plane spacing with respect to α-GeTe during the first 200 seconds of growth.

**Figure 2:**
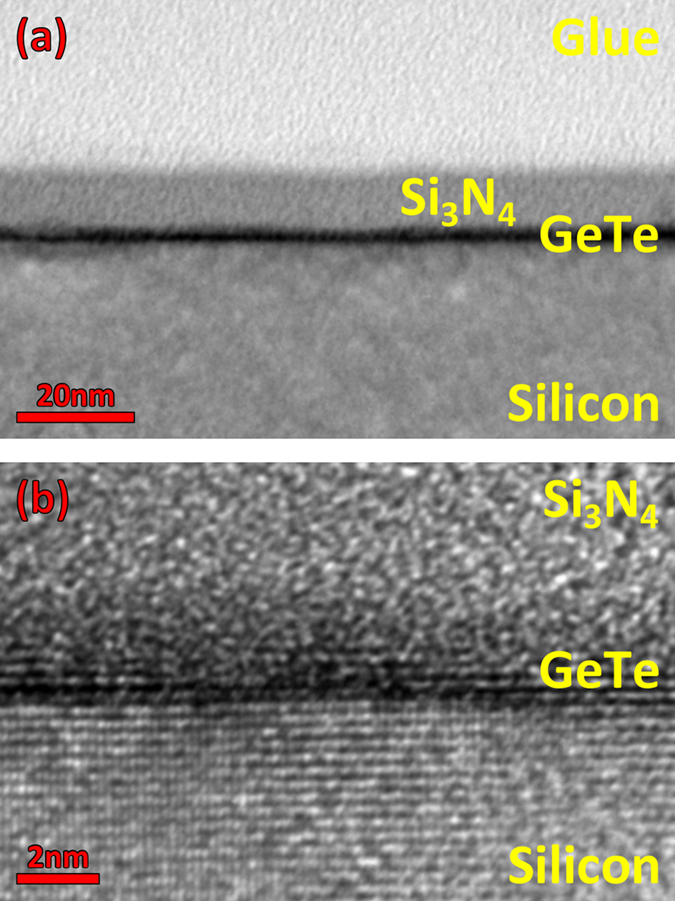
(**a**) Low resolution TEM micrograph of a nominal 1 BL thick GeTe film grown on Si(111)-(√3 × √3)R30°-Sb. (**b**) Cross-section HRTEM along Si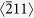 on the same sample as in (**a**). These micrographs show that GeTe forms a crystalline and wetting film. The simultaneous coalescence of more than one layer at the same time is also observed.

**Figure 3:**
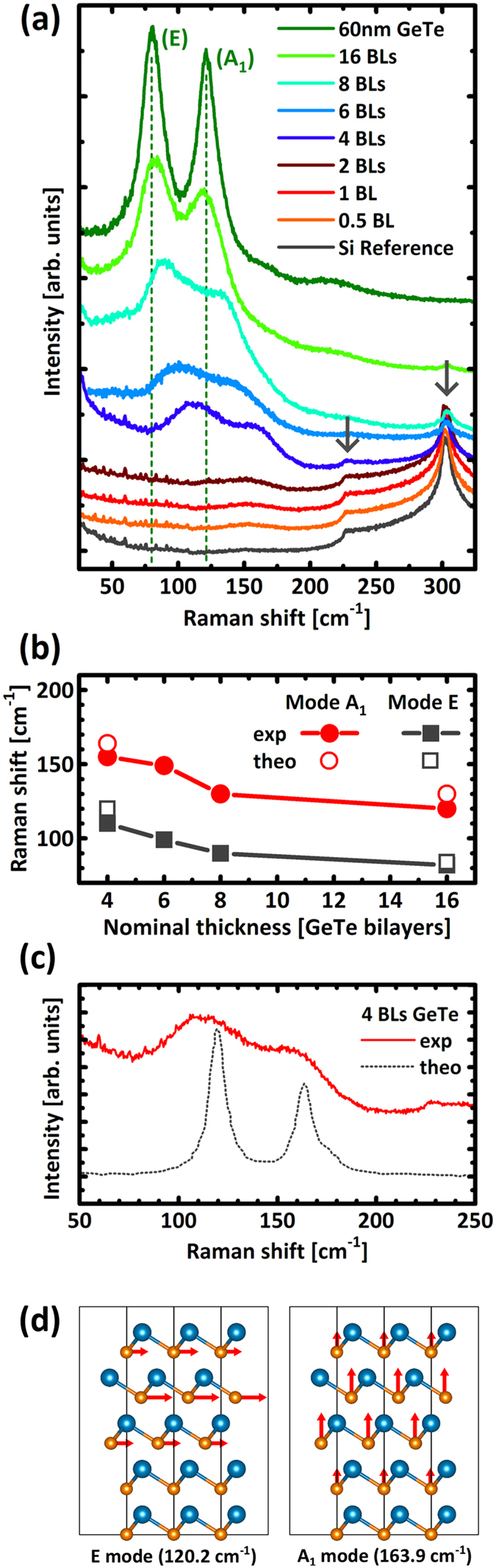
(**a**) Raman spectra acquired on GeTe samples of increasing thickness of 0.5, 1, 2, 4, 6, 8. 16 BLs grown on Si(111)-(√3 × √3)R30°-Sb. Measurements on a silicon reference and a thicker 60 nm GeTe film are shown as a comparison. (**b**) GeTe (A_1_) and (E) mode strengthening with decreasing film thickness, calculated values for 4 BL and bulk are plotted with open symbols for comparison. (**c**) Raman spectrum acquired on 4 BL GeTe sample grown on Si(111)-(√3 × √3)R30°-Sb (line) compared with an analogous spectrum calculated by DFT at 0 K (dashed line). GeTe (A_1_) and (E) modes are visible. (**d**) Displacement patterns for the two active Raman modes (E left, A_1_ right) of the 4 BL supported on the bulk.
