# Supplementary material for: Ordered Peierls distortion prevented at growth onset of GeTe ultra-thin films
Source: Sci Rep. 2016 Sep 9;6:32895. doi: 10.1038/srep32895 (PMC5017194; doi:10.1038/srep32895)
Supplement: Supplementary Information [file srep32895-s1.pdf]

Ordered Peierls distortion prevented at growth onset of GeTe ultra-thin films

*Ruining Wang [1], Davide Campi [2], Marco Bernasconi [2], Jamo Momand [3], Bart J. Kooi [3], Marcel A. Verheijen,[4] Matthias Wuttig,[5,6] Raffaella Calarco [1]**

[1] Paul-Drude-Institut für Festkörperelektronik, Hausvogteiplatz 5-7, 10117 Berlin, Germany

[2] Department of Materials Science, University of Milano-Bicocca via R. Cozzi 55, 20125 Milano, Italy

[3] Zernike Institute for Advanced Materials, University of Groningen, Nijenborgh 4, 9747 AG Groningen, The Netherlands

[4] Eindhoven University of Technology, Department of Applied Physics, NL-5600 MB Eindhoven, The Netherlands

[5] I. Physikalisches Institut, RWTH Aachen University, 52056 Aachen, Germany

[6] JARA-FIT and JARA-HPC, RWTH Aachen University, 52056 Aachen, Germany

KEYWORDS: epitaxy, coincidence lattices, surface reconstructions, phase change materials, resonant bonding, Peierls distortion.

SUPPLEMENTARY INFORMATION

RHEED study of GeTe growth onset on Si(111)-(√3×√3)R30-Sb at low Ge flux

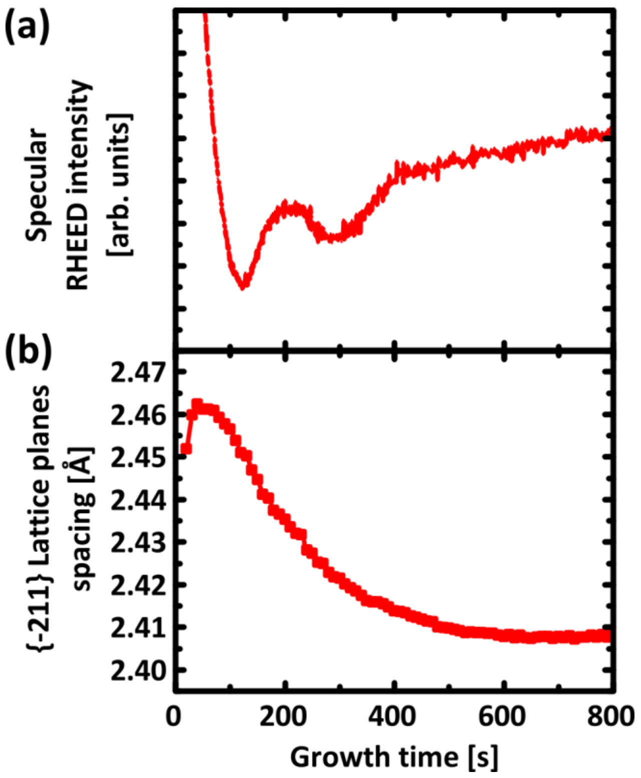

Figure S1 – *(a) Specular beam intensity oscillations close to growth onset in the case of a half Ge flux growth. (b) {-211} lattice planes spacing calculated from the RHEED streak spacing.*

In order to rule out the possibility for the phenomenon observed to be a kinetic effect, a similar deposition was performed with a reduced growth rate; with a Ge flux reduced by ~50%. Indeed, as shown in Figure S1(a), the period of the two first specular RHEED intensity oscillations doubled. The first minimum was found at t = 100 s, the first maximum at t = 200 s, and second maximum at t = 400 s. Exactly the same changes in lattice spacing aforementioned were also observed in this case: As shown in Figure S1(b), the in-plane lattice spacing immediately at growth onset is calculated to be 2.46 Å, and within the two first

RHEED intensity oscillations, it is lowered to a value of 2.41 Å, which corresponds to the expected value for bulk α-GeTe. Therefore it is concluded that the changes observed at growth onset are thermodynamically driven, and not due to growth kinetics.

Raman spectra magnified in the 100-200 cm^{-1} shift range for 0.5, 1, and 2 BL samples

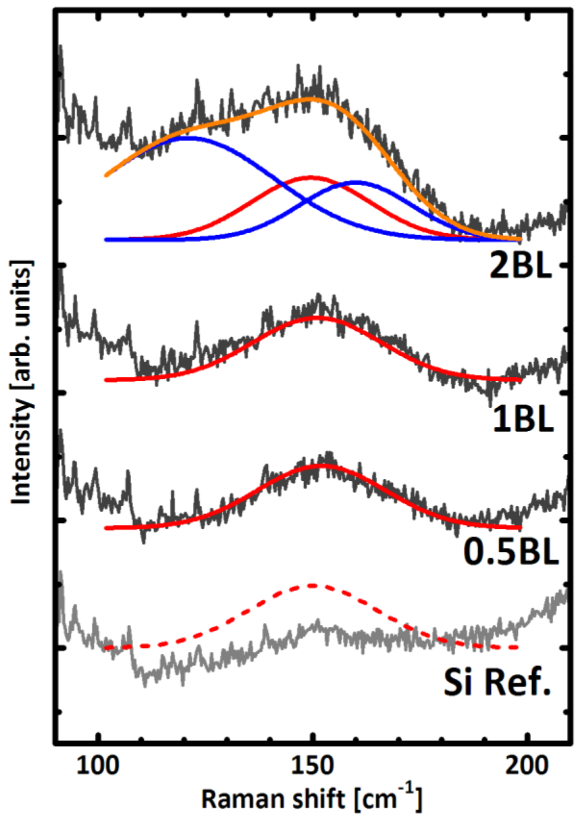

Figure S2 – *Raman spectra for 0.5, 1, and 2 BL samples fitted using Guaussian functions. The red curve shows the Raman feature originating from the Sb-passivation. The two blue curves represent modes from GeTe, and the orange line shows the cumulative fitted curve.*

The Raman features in the 2 BL sample are best resolved after fitting by Guaussian functions in a magnified viewgraph. The contribution from the Sb-passivation is shown by the red curves; it is the only feature present in the 0.5 and 1 BL spectra. The 2 BL sample clearly yields additional modes that can be ascribed to GeTe, represented by blue curves. These modes positioned at 120 and 160 cm^{-1} are consistent with the (E) and (A1) modes

strengthening described in Figure 3(b), the relative intensity between the two modes is also well respected. A comparison with the silicon reference with no surface preparation is also shown (native oxide surface); the feature stemming from the Sb-passivation is of course absent.

STEM-HAADF images on ultra-thin GeTe films

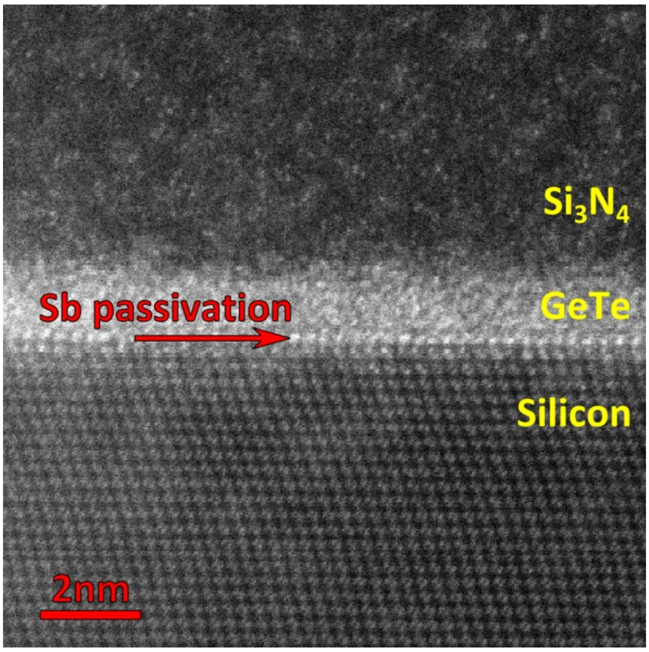

Figure S3 – *STEM-HAADF micrograph of a nominal 1 BL thick GeTe film grown on Si(111)-(√3×√3)R30°-Sb. The row of atoms in brighter contrast at the interface are ascribed to the Sb atoms passivating the silicon substrate.*

Ongoing STEM investigations highlight that the designed Sb passivation of the initial Si(111) surface is preserved after the deposition of the GeTe layer. Thus, intermixing can be ruled out as the origin for the larger than expected in-plane lattice spacing observed in RHEED (Figure 1(d)). The crystalline order in the GeTe film itself has been perturbed on this micrograph, and the most probable cause is prolonged exposure to ambient air, causing GeTe to oxidize. [1,2] The Raman data presented in Figure 3(a) demonstrate that the film was not amorphous after growth and deposition of the capping layer. Even an ultra-thin layer of

amorphous material would immediately yield Raman features associated to the hybridized tetragonal Ge-Te bonds.[3] A Bose peak in the range of low frequency shifts would also be observed.[4] Therefore, it is determined that the sample was altered at some point in the timeframe after growth and before imaging.

Detailed description of the DFT calculations:

We computed the Raman spectrum from phonons at the Γ point within density functional perturbation theory (DFPT). [5] The differential cross section for Raman scattering (Stokes) in non-resonant conditions is computed from phonons and the derivatives of the static electronic susceptibility with respect to atomic displacements as described in our previous works. [6]

In the article we report the theoretical Raman spectrum and the frequency of A_1 and E modes for a thick slab of GeTe with a numbers of layers free to move and few bottom layers frozen mimicking the surface substrate. The layers free to move are equal in number to those of the multilayers grown experimentally (1 - 4 BL). The in-plane lattice parameter is fixed to the theoretical bulk value as obtained in our previous work. [7] For multilayers thinner than the 4 BL we also considered the in-plane lattice parameters fixed to the experimental value as measured during the MBE growth. Equilibrium geometries were obtained by optimizing the internal structure at the fixed in-plane lattice parameters.

Moreover we also considered free standing multilayers, i.e. without the frozen layers at the bottom, with different choices of the in-plane lattice parameters: the bulk theoretical ones (a = 4.208 Å and c = 10.749 Å in the hexagonal setting), the experimental ones for the multilayers, or the theoretical ones obtained by fully relaxing the slab geometries.

In the multilayers there are several E and A_1 modes ideally corresponding to the folding at Γ of bulk-like phonon branches along the c axis of the multilayers. The mode with the largest

Raman activity has actually the lower frequency for both the A_1 and E modes due to the upward curvature of the phonon bands from the Γ point along the c axis (see for instance [7]).

The phonon frequency of the most intense Raman mode for the multilayers with different choices of the in-plane lattice parameters are reported in Tables S1-3. The in-plane lattice parameter shrinks sizably with respect to the bulk which leads to an enhancing of the difference between short and long (resonating) bonds with respect to the bulk.

	d_s (Å)	d_l (Å)	E mode (cm^{-1})	A_1 mode (cm^{-1})
1 BL	2.65	-	148	198
2 BL	2.82	3.31	141	188
4 BL	2.82	3.31	132	175

Table S1 – *Frequency of the main Raman active A_1 and E modes for free-standing multilayers with bulk in-plane lattice parameters. d_s and d_l are respectively the lengths of the short and long Ge-Te bonds to be compared with the theoretical values of d_s=2.85 and d_l=3.21Å in the bulk.*

	a_h (Å)	d_s (Å)	d_l (Å)	E mode (cm^{-1})	A_1 mode (cm^{-1})
1 BL	3.93	2.75	-	166	226
2 BL	4.05	2.79	3.32	155	205
4 BL	4.12	2.82	3.26	140	184

Table S2 – *Frequency of the main Raman active A_1 and E modes for free-standing multilayers with optimized lattice parameters. a_h is the in-plane lattice parameter in the hexagonal setting.*

	a_h (Å)	d_s (Å)	d_l (Å)	E mode (cm^{-1})	A_1 mode (cm^{-1})
1 BL	4.26	2.85	-	144	191
2 BL	4.26	2.83	3.32	136	181

Table S3 – *Frequency of the main Raman active A_1 and E modes for free-standing multilayers with in-plane lattice parameters fixed to experimental one for the GeTe monolayer.*

REFERENCES

1. Gourvest, E. *et al.* Impact of Oxidation on Ge2Sb2Te5 and GeTe Phase-Change Properties. *J. Electrochem. Soc.* **159,** H373 (2012).

2. Yashina, L. V., Kobeleva, S. P., Shatalova, T. B., Zlomanov, V. P. & Shtanov, V. I. XPS study of fresh and oxidized GeTe and (Ge,Sn)Te surface. *Solid State Ionics* **141-142,** 513–522 (2001).

3. Andrikopoulos, K. S. *et al.* Raman scattering study of the a-GeTe structure and possible mechanism for the amorphous to crystal transition. *J. Phys. Condens. Matter* **18,** 965–979 (2006).

4. Andrikopoulos, K. S., Yannopoulos, S. N., Kolobov, A. V., Fons, P. & Tominaga, J. Raman scattering study of GeTe and Ge2Sb2Te5 phase-change materials. *J. Phys. Chem. Solids* **68,** 1074–1078 (2007).

5. Baroni, S., De Gironcoli, S., Dal Corso, A. & Giannozzi, P. Phonons and related crystal properties from density-functional perturbation theory. *Rev. Mod. Phys.* **73,**

515–562 (2001).

6. Sosso, G. C., Caravati, S. & Bernasconi, M. Vibrational properties of crystalline Sb(2)Te(3) from first principles. *J. Phys. Condens. Matter* **21,** 095410 (2009).

7. Campi, D., Donadio, D., Sosso, G. C., Behler, J. & Bernasconi, M. Electron-phonon interaction and thermal boundary resistance at the crystal-amorphous interface of the phase change compound GeTe. *J. Appl. Phys.* **117,** 015304 (2015).
